# Carotenoids retention in biofortified yellow cassava processed with traditional African methods

**DOI:** 10.1002/jsfa.9347

**Published:** 2018-10-29

**Authors:** Victor Taleon, Dan Sumbu, Tawanda Muzhingi, Sylvain Bidiaka

**Affiliations:** ^1^ HarvestPlus, c/o International Food Policy Research Institute (IFPRI) Washington DC USA; ^2^ HarvestPlus, c/o International Institute of Tropical Agriculture (IITA) Kinshasa Congo; ^3^ CIP‐SSA Regional Office, Food and Nutrition Evaluation Laboratory Biosciences for Eastern and Central Africa (BecA) Nairobi Kenya

**Keywords:** biofortification, yellow cassava, carotenoid retention, *chikwangue*, *fufu*

## Abstract

**BACKGROUND:**

Biofortified yellow cassava is being cultivated in countries with high cassava consumption to improve its population's vitamin A status. The carotenoid retention in biofortified cassava when processed as boiled, *fufu*, and *chikwangue* was evaluated in this study. Commercial biofortified varieties Kindisa and Vuvu and the experimental genotypes MVZ2011B/360 and MVZ2012/044 were used. Fresh cassava roots were processed as boiled, *fufu*, and *chikwangue*. Provitamin A carotenoids (pVACs) content of fresh and processed cassava was measured by high‐performance liquid chromatography, and total carotenoids was measured by spectrophotometer.

**RESULTS:**

pVACs content of fresh peeled cassava was 1.79–6.65 µg g^−1^ on a fresh weight basis, whereas in boiled cassava, *fufu*, and *chikwangue* the pVACs content was 1.71–6.91 µg g^−1^, 0.04–0.37 µg g^−1^, and 0.52–1.75 µg g^−1^ respectively. True retention of carotenoids after cooking was 93.2–96.8%, 0.8–3.1%, and 4.0–18.1% for boiled cassava, *fufu*, and *chikwangue* respectively. Significant total carotenoids loss was observed during storage.

**CONCLUSION:**

The results indicated that biofortified boiled cassava could be an effective food product to improve pVACs intake in areas where vitamin A deficiency exists, and processing of *chikwangue* and *fufu* should be improved before promoting biofortified cassava in vitamin‐A‐deficient areas with high cassava consumption. © 2018 The Authors. *Journal of the Science of Food and Agriculture* published by John Wiley & Sons Ltd on behalf of Society of Chemical Industry.

## INTRODUCTION

Vitamin A deficiency, which is associated with risk of infections and xerophthalmia, is a severe public health problem in countries like Nigeria, Democratic Republic of Congo (DRC) and the Republic of the Congo, where cassava is the main source of carbohydrates.^1^, ^2^ Biofortified staple crops with higher micronutrient density, including varieties of biofortified yellow cassava with provitamin A carotenoids (pVACs), have been developed to contribute reducing micronutrient deficiencies across the world.^3^ Biofortified cassava varieties developed by conventional breeding techniques have been released in the main cassava‐producing countries, such as the DRC and Nigeria. In 2008, the first biofortified cassava variety (Kindisa), with pVACs levels between 6 and 8 µg g^−1^ *β*‐carotene equivalents (*β*CE), was released in the DRC. Varieties of biofortified cassava with higher pVACs content (target level of 15 µg g^−1^ *β*CE) are being developed by the Institut National pour l'Etude et la Recherche Agronomiques (INERA) in the DRC and the Nigerian National Root Crops Research Institute in Nigeria. The pVACs target level for cassava, set to reach 50% of the estimated average requirement (EAR) for children and pregnant women in the DRC and Nigeria, assumes that up to 50% of pVACs in peeled roots is lost during processing, storage, and cooking.^4^, ^5^

Carotenoid retention higher than 50% in boiled cassava has been reported in different studies.^6^, ^7^ A study in Kenya demonstrated that feeding children 2–4 years old with boiled biofortified yellow cassava improved their vitamin A status.^8^ However, commonly consumed cassava‐derived food products in African countries, such as *fufu*, *gari*, *chikwangue*, and *lafun*, are produced with fermented cassava roots to eliminate most of the toxic cyanogenic compounds present in raw cassava.^9^ Cassava is mainly commercialized either as dry pieces of fermented cassava roots, *cossettes*, that are milled into cassava flour to prepare *fufu* or *lafun*, or as fermented cassava paste, *kimpuka*, used to prepare *chikwangue*. Generally, *fufu* is prepared by cooking fermented cassava flour in boiling water, whereas *chikwangue* is prepared by precooking and steaming fermented cassava paste.^10^, ^11^, ^12^, ^13^ Limited information on carotenoid retention is available for both products, making it difficult to estimate their potential impact on vitamin A status of children and women. In Nigeria, a study found that apparent carotenoid retention in *fufu* prepared with fermented cassava flour was 17–32%, but no information on true retention was presented.^14^ The same study also found that apparent retention of carotenoids was 86–90% when *fufu* was prepared with a wet paste without a drying step. Another study in Nigeria reported true carotenoid retention between 12 and 36% when processing biofortified cassava roots into *fufu* using fermented cassava paste without a drying step.^15^ Furthermore, cassava chips to prepare *fufu* and paste to prepare *chikwangue* are transported for long distances and stored during extended periods up to 1 month, which could affect the final pVACs of cooked products made with them.

To determine the potential contribution of biofortified cassava to vitamin A EAR of populations with high consumption of cassava, this study measured the retention of carotenoids during the processing of biofortified cassava into boiled cassava, *fufu*, and *chikwangue*.

## MATERIALS AND METHODS

### Cassava roots and food products

Two released biofortified genotypes of cassava, Kindisa and Vuvu, and two yellow advanced experimental genotypes, MVZ2011B/360 and MVZ2012/044, were used to determine the carotenoid retention during boiling and production of *fufu* and *chikwangue*. The materials were selected based on their root color and acceptable root yield in breeding fields. Cassava was grown under conventional agronomic practices in February 2015 at the INERA station in Mvuazi, Kongo Central, and harvested in April 2016. Roots from each genotype were harvested in batches of 70 kg per processing repetition. Harvesting of each batch was done on different days and the processing of each batch started the day of harvest. From each batch, 10 kg was used to produce boiled cassava, 30 kg to produce *fufu*, and 30 kg to produce *chikwangue*. In addition, to determine carotenoid retention during storage of fermented cassava paste and chips, 420 kg of roots (Kindisa) was harvested and distributed among seven cassava processors across Kongo Central to produce fermented cassava paste and chips.

### Processing of boiled cassava

A 10 kg batch of fresh cassava roots was peeled manually, and chopped into pieces of 8–10 cm length and 3–5 cm width and height. A 2 kg sample of the peeled cassava pieces was boiled for 40 min at >98 °C in a pot containing 4 L of boiling water, using an electric stove. Raw and cooked cassava were weighed and a sample of each was taken for measurement of total carotenoids (TCs), pVACs, and dry matter content.

### Processing of *fufu*

A 30 kg batch of cassava roots was washed, manually peeled, and chipped using an AGRIMAC mechanical chipper (Kinshasa, DRC) with a cutting disc to produce small cassava chips, *microcossettes*, of approximately 25 mm length, 5 mm width, and 2 mm height. Chipped cassava was transferred into a polypropylene sack and fermented for 3 days inside a plastic bucket containing 27 L of water and covered with a dark plastic to prevent direct exposure to light. After fermentation (pH <4.0), cassava was pressed four times in a hydraulic press (5 min each) to remove excess water. Pressed cassava was sundried for 3 days (4 h per day) to obtain fermented cassava chips with 9.8–11.0% moisture. The fermented *microcossettes* were milled using a hammer mill to obtain fermented cassava flour with particle size <0.5 mm. The freshly milled fermented cassava flour was mixed for 4 min with boiling water in a 1 : 3–1 : 4 ratio with a wooden spatula until obtaining a uniform cooked paste called *fufu*. Samples of pressed fermented cassava (paste), milled *microcossettes* (flour), and cooked *fufu* were taken for measurement of TCs, pVACs, and dry matter content.

### Processing of *chikwangue*

A 30 kg batch of cassava roots was washed, peeled manually, and grated using an AGRIMAC mechanical grater (Kinshasa, DRC). Grated cassava was put in a polypropylene sack and fermented for 4 days inside a plastic bucket containing 27 L of water and covered as noted earlier. After fermentation (pH 3.6–3.8), water was drained, roots were smashed by hand, and large pieces of fibers were removed, resulting in a fermented cassava paste, also named *kimpuka*. The *kimpuka* was sieved with excess water to remove small pieces of fiber residue, sedimented, decanted to remove free water, drained in polypropylene sacks for 16 h to remove excess water, and finally pounded to obtain a uniform fine paste. The fine paste was placed inside 1 kg plastic bags and partially cooked in boiling water for 20 min. The partially cooked paste was kneaded to form a dough and molded into pieces of approximately 700 g, placed inside polyethylene bags that were wrapped in leaves (*Megaphrynium marostachyum*), and finally steamed for 40 min to obtain the cooked *chikwangues*. Samples of the fine paste, cooked *chikwangues*, and fiber residue were taken for measurement of TCs, pVACs, and dry matter content.

### Cassava flour and paste for storage

Samples of *microcossettes* and *kimpuka* prepared with biofortified cassava variety Kindisa were collected from seven processing centers across Kongo Central province to determine retention of TCs during storage. Samples were collected immediately after the processing operations ended at each of the processing centers and transported inside an insulated foam container to a storage facility within 24 h. The fermented *microcossettes* and paste collected were stored at room temperature (24–28 °C, daily average) in a dark room for 20 days and 30 days respectively. The *microcossettes* were stored in transparent polyethylene bags and samples were taken at days 0, 10, and 20 of storage for TC analysis, whereas the paste was stored in the same bag type and samples were taken at days 0, 10, 20, and 30 of storage.

### Reagents for carotenoid extraction and quantification

All reagents and chemical used were of high‐performance liquid chromatography (HPLC) and analytical grade. The all‐*trans*‐*β*‐carotene (*β*C), 9‐*cis*‐*β*‐carotene (9‐*cis*‐*β*C), and 13‐*cis*‐*β*‐carotene (13‐*cis*‐*β*C) standards were purchased from CaroteNature (Ostermundigen, Switzerland). Butylated hydroxytoluene, methyl‐*tert*‐butyl ether, hexane, ethanol, methanol, and ammonium acetate were obtained from Sigma Co. (St Louis, MO, USA).

### TCs measurement by spectrophotometer

The extraction method described by Rodriguez‐Amaya with some modifications was used to measure TCs using a spectrophotometer.^16^ Each sample was homogenized and 10 g was mixed with 3 g of Celite and 25 mL of acetone. The mixture was pounded with a pestle for 3 min and transferred to a filter funnel. To ensure complete removal of carotenoids from the filter funnel, an additional 30 mL of acetone was added. Filtered extract was placed into a 500 mL separatory funnel containing 40 mL of petroleum ether. Acetone was separated from the petroleum ether by slowly adding 50 mL of deionized water. To further remove acetone from the petroleum ether phase, 50 mL of water (10 g kg^−1^ sodium chloride) was added four times. The resulting extract was filtered with 15 g of anhydrous sodium sulfate and placed into a 50 mL graduated flask. Petroleum ether was added until carotenoid extract was diluted to 50 mL. An aliquot of the extract was placed in a quartz cuvette and was read at 450 nm using a Genesys 10S UV–visible spectrophotometer. To calculate the TCs content, the following equation was used:

$$\mathrm{TC}=\frac{A\times V\times 10^{-4}}{A_{1\;\mathrm{cm}}^{1\%}\times P}$$

where TC (µg g^−1^) is the TCs in the sample, *A* is the absorbance at 450 nm; *V* (mL) is the final volume of extract, $A_{1\;\mathrm{cm}}^{1\%}=2592$ is the molar absorptivity coefficient of *β*‐carotene in petroleum ether), and *P* (g) is the weight of sample.

### Carotenoid extraction and measurement by HPLC

Samples were analyzed at the Food and Nutritional Evaluation Laboratory at the International Potato Center (CIP), BecA ILRI Research Hub, Nairobi, Kenya. Carotenoid extraction and analysis was performed as described by Kurilich and Juvik, with some modifications.^17^ Samples were equilibrated to room conditions and 1 g was weighed in duplicate in 25 mL glass tubes; 6 mL of ethanol with 1 g kg^−1^ butylated hydroxytoluene was added to the tube, vortexed for 1 min, and incubated in a hot‐water bath at 85 °C for 10 min. Samples were saponified by adding 120 µL of 14 mol L^–1^ potassium hydroxide into the tubes, vortexing for 1 min and incubating in a water bath at 85 °C for 5 min. After saponification, the tubes were immediately placed in an ice bath and 3 mL of cold deionized distilled water was added. Hexane (5 mL) was added to each tube and vortexed for 1 min and centrifuged at 800×*g* for 10 min. The upper phase of hexane was pipetted into a 25 mL test tube. The hexane extraction was repeated three more times on the residue. The combined hexane fractions were washed by adding 4 mL of deionized water, vortexing, centrifuging at 800×*g* for 5 min and the upper layer containing the hexane fractions transferred into separate test tubes. The hexane fractions were dried to complete dryness under a gentle stream of nitrogen using an Organomation N‐Evap System (Organomation, Berlin, MA, USA). The residue was reconstituted in 5 mL of ethanol and immediately vortexed and sonicated for 30 s. A 1.5 mL aliquot was transferred into a 2 mL HPLC vial from which 50 µL was taken for HPLC analysis. The carotenoids were separated using a YMC C30 column, 3 µm, 150 mm × 4.6 mm column (YMC Europe GMBH, Dinslaken, Germany) in an Alliance 2695 HPLC system (Waters, Milford, MA, USA). The flow rate was 1 mL min^−1^ by a gradient elution with two mixtures of methanol, methyl‐*tert*‐butyl ether, and water (mixture A: 83/15/2 (v/v/v); mixture B: 8/90/2 (v/v/v); both solvents with 15 g kg^−1^ ammonium acetate). The gradient time program was 0–1 min 100% A, 1–8 min linear gradient to 70% A, 8–13 min 70% A, 13–22 min linear gradient to 45% A, 22–24 min 45% A, 24–34 min linear gradient to 5% A, 34–38 min 5% A, 38–40 min linear gradient to 100% A, and 40–50 min 100% A. A Waters 2996 photodiode array detector at 450 nm and Waters Empower 2 software were used to identify and quantify the carotenoids from extracts by determining the peak area of extracts and standard dilutions.

### Apparent and true carotenoid retention

For all samples, percentage of true retention (TR) and apparent retention (AR) were calculated as follows:

$$\begin{array}{cc} \mathrm{TR}\;\left(\%\right)= & \frac{\begin{array}{c} \text{pVACs}/g\;\text{processed or stored cassava}\;\\ \left(\mathrm{dry}\;\text{basis}\right)\times g\;\text{cassava after process or storage} \end{array}}{\begin{array}{c} \text{pVACs}/g\;\text{cassava before processing or storage}\\ \;\left(\mathrm{dry}\;\text{basis}\right)\times g\;\text{cassava before processing or storage} \end{array}}\\ & \times 100 \end{array}$$

$$\begin{array}{cc} \mathrm{AR}\;\left(\%\right)= & \frac{\begin{array}{c} \text{pVACs}/g\;\text{processed or stored product}\\ \;\left(\mathrm{dry}\;\text{basis}\right) \end{array}}{\begin{array}{c} \text{pVACs}/g\;\text{cassava before processing or storage}\\ \;\left(\mathrm{dry}\;\text{basis}\right) \end{array}}\\ & \times 100 \end{array}$$

TR percentage values were used to determine the total proportion of carotenoids lost during processing, storage, and cooking. AR percentage values were used to determine the carotenoid concentration in intermediate or final (cooked) products without considering losses of solids during processing, storage, or cooking. AR is useful to understand the contribution of a processed product to vitamin A intake based on the intake of such product. TR is useful to understand the contribution of a crop to vitamin A intake based on the expected amount of crop available per person.

### Data analysis

Data were expressed as mean plus/minus standard deviation of three processing repetitions. The effect of processing step and genotype was evaluated using a two‐factor analysis of variance model for each cassava food product. Each repetition was represented by a different processing day. Analysis of variance was analyzed using the mixed procedure in SAS 9.4 (SAS Institute, Cary, NC, USA). Means separation was calculated using Tukey's method. Differences between means were considered significant at *p* < 0.05. The total pVACs was expressed as *β*CE and was calculated as follows: *β*CE = *β*C + (0.5)(9‐*cis*‐*β*C) + (0.5)(9‐*cis*‐*β*C). The conversion factor of *β*CE to retinol equivalent to calculate contribution to vitamin A EAR was 5 : 1.^18^

Rate of carotenoid degradation *k* was calculated as follows:

$\ln\frac{x}{x_{0}}=-\mathrm{kt}$

where *x* is the concentration of carotenoid at time *t* (number of days in storage), *x*_0_ is the concentration of carotenoid at the beginning of storage, and *k* (day^−1^) is the reaction rate constant.

## RESULTS AND DISCUSSION

### Carotenoid profile of cassava roots

Changes in the TCs and pVACs ranking among the four genotypes were found when expressed as fresh weight (FW) basis compared with dry weight (DW) basis (Table 1). For this reason, the carotenoid concentration was expressed in FW and DW basis for peeled cassava and final cooked cassava products; for all other intermediate steps, the concentration values were presented in DW only. TCs content of peeled cassava roots measured by UV–visible spectrophotometer was 2.35–6.86 µg g^−1^ FW and 5.21–25.80 µg g^−1^ DW. Total pVACs content quantified by HPLC was 1.79–6.65 µg g^−1^ FW and 3.96–25.02 µg g^−1^ DW, where MVZ2011B/360 had the highest pVACs content and Vuvu the lowest. In all genotypes, the main carotenoid found in peeled cassava roots was *β*C, whereas 9‐*cis*‐*β*C and 13‐*cis*‐*β*C were found in lower concentrations (Table 1). The proportions of *β*C, 9‐*cis*‐*β*C, and 13‐*cis*‐*β*C isomers were respectively 74.8%, 9.1% and 16.0% of the total *β*‐carotene identified in fresh cassava roots. These values are within the range reported by Thakkar *et al*.^19^ and Ceballos *et al*.^20^ in which *β*C represented 55–85% of the total *β*‐carotene isomers and 13‐*cis*‐*β*C and 9‐*cis*‐*β*C represented 15–45%. The pVACs in fresh roots represented 11.9–44.3% of the 15 µg g^−1^ *β*CE target value for biofortified cassava.

**Table 1:** Carotenoid content in fresh roots of four yellow cassava genotypes

	Fresh weight basis (µg g^−1^ ± SD)					Dry weight basis (µg g^−1^ ± SD)	
Genotype	TCs	13‐*cis*‐*β*C	*β*C	9‐*cis*‐*β*C	pVACs	TCs	pVACs
MVZ2011B/360	6.86 ± 0.09^a^	1.12 ± 0.05^a^	5.87 ± 0.20^a^	0.43 ± 0.02^b^	6.65 ± 0.23^a^	25.80 ± 0.94^a^	25.02 ± 1.75^a^
MVZ2012/044	4.69 ± 0.07^c^	0.81 ± 0.04^c^	3.54 ± 0.03^c^	0.39 ± 0.05^b^	4.15 ± 0.07^c^	22.42 ± 0.60^b^	19.84 ± 0.83^b^
Kindisa	5.58 ± 0.33^b^	0.99 ± 0.01^b^	4.95 ± 0.27^b^	0.63 ± 0.03^a^	5.76 ± 0.29^b^	15.38 ± 1.00^c^	15.87 ± 0.95^c^
Vuvu	2.35 ± 0.01^d^	0.45 ± 0.01^d^	1.33 ± 0.06^d^	0.46 ± 0.02^b^	1.79 ± 0.06^d^	5.21 ± 0.11^d^	3.96 ± 0.14^d^

Different lowercase letters indicate significant differences within columns (*p* < 0.05).

Values are average plus/minus standard deviation (SD) of three processing replicates. 13‐*cis*‐*β*C = 13‐*cis*‐*β*‐carotene; 9‐*cis*‐*β*C = 9‐*cis*‐*β*‐carotene; *β*C = all‐*trans*‐*β*‐carotene; pVACs, provitamin A carotenoids expressed as *β*‐carotene equivalents; TCs, total carotenoids measured using UV–visible spectrophotometer.

### Carotenoid retention in boiled cassava

The pVACs TR values after boiling cassava roots were 93.2–96.8%, whereas AR values were 97.0–102.6% (Table 2). No significant differences were found in TR and AR between genotypes (*p* > 0.05). These retention values were similar to AR >100.0% found by Thakkar *et al*.^19^ for three varieties of biofortified yellow cassava, and within the TR (76.0–96.7%) reported by Ceballos *et al*.^20^ for six biofortified yellow cassava genotypes. Total pVACs after boiling was 1.71–6.91 µg g^−1^ FW and 3.90–25.63 µg g^−1^ DW (Table 2). As in fresh roots, boiled roots of MVZ2011B/360 had the highest pVACs content and Vuvu had the lowest. The main carotenoid found after boiling cassava was *β*C, representing 72.1% of total quantified carotenoids, whereas 13‐*cis‐β*C and 9‐*cis‐β*C represented 8.1% and 19.7% respectively. The proportions of *β*‐carotene isomers after boiling were similar to those in fresh roots, suggesting that no significant isomerization of *β*C to 13‐*cis‐β*C or 9‐*cis‐β*C occurred during boiling for these genotypes. Significant isomerization of *β*C to 13‐*cis‐β*C was observed by Thakkar *et al*.^19^ when boiling cassava previously soaked in water for at least 10 h. No isomerization of *β*C to 9‐*cis‐β*C or isomerization to 13‐*cis‐β*C instead of 9‐*cis β*‐carotene could be positive considering that 13‐*cis‐β*C has shown better bioconversion to retinol than 9‐*cis‐β*C has.^21^ The very high carotenoid retention during boiling yellow cassava makes it a potential alternative to deliver pVACs to consumers. Before promoting cassava consumption as boiled, the cyanogenic content should be evaluated because it is known that cassava could contain high levels of cyanide if not cooked properly.^22^

**Table 2:** Provitamin A carotenoids and carotenoid retention in four yellow cassava genotypes after different processing methods

Process type	Genotype	pVACs FW (µg g^−1^ ± SD)^a^	pVACs DW (µg g^−1^ ± SD)^a^	True retention (% ± SD)^a^	Apparent retention (% ± SD)^a^
Boiled	MVZ2011B/360	6.91 ± 0.21^a^	25.63 ± 1.65^a^	96.8 ± 4.5^a^	102.6 ± 5.5^a^
	MVZ2012/044	4.36 ± 0.18^c^	19.43 ± 0.83^b^	96.4 ± 5.2^a^	98.0 ± 5.4^a^
	Kindisa	5.80 ± 0.78^b^	15.42 ± 2.01^c^	93.2 ± 9.0^a^	97.0 ± 8.4^a^
	Vuvu	1.71 ± 0.19^d^	3.90 ± 0.41^d^	94.9 ± 6.6^a^	98.4 ± 6.9^a^
*Chikangue*	MVZ2011B/360	1.75 ± 0.62^a^	6.73 ± 2.35^a^	5.6 ± 0.9^b^	26.3 ± 6.8^a^
	MVZ2012/044	1.21 ± 0.24^a^	4.28 ± 0.92^a^	4.0 ± 0.4^b^	21.6 ± 4.8^a^
	Kindisa	1.50 ± 1.24^a^	4.69 ± 3.90^ab^	8.10 ± 6.2^b^	28.8 ± 22.4^a^
	Vuvu	0.39 ± 0.23^b^	1.04 ± 0.62^b^	18.1 ± 1.7^a^	35.7 ± 4.7^a^
*Fufu*	MVZ2011B/360	0.22 ± 0.17^ab^	0.63 ± 0.47^b^	1.8 ± 1.2^ab^	2.5 ± 1.7^b^
	MVZ2012/044	0.37 ± 0.13^a^	1.11 ± 0.38^a^	3.1 ± 1.4^a^	5.6 ± 1.9^a^
	Kindisa	0.08 ± 0.04^b^	0.23 ± 0.11^b^	0.8 ± 0.5^b^	1.5 ± 0.7^b^
	Vuvu	0.04 ± 0.03^b^	0.13 ± 0.09^b^	2.5 ± 1.7^ab^	3.3 ± 2.3^b^

Different lowercase letters indicate significant differences within columns for each process type (p < 0.05).

Values are average plus/minus standard deviation (SD) of three processing replicates. DW, dry weight basis; FW, fresh weight basis; pVACs, provitamin A carotenoids expressed as *β*‐carotene equivalents.

### Carotenoid retention in cooked *fufu*

**Figure 1:**
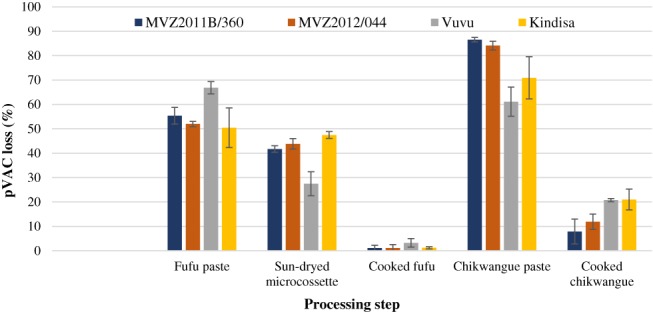
Loss of pVACs (%) in each processing step of three cassava products (boiled, *fufu*, and *chikwangue*) made with four yellow biofortified cassava varieties.

The TR of pVACs in cooked *fufu* prepared with freshly fermented *microcossettes* was 0.8–3.1%, whereas AR was 1.5–5.6% (Table 2). The pVACs in cooked *fufu* was 0.04–0.37 µg g^−1^ FW and 0.23–1.11 µg g^−1^ DW. Genotype MVZ2012/044 had higher TR than Kindisa did, but no significant differences were found with the other genotypes. MVZ2012/044 had the highest pVACs AR among the four genotypes. The TR values in *fufu* were lower than the 12–36% TR reported for *fufu* produced with nondried fermented paste in Nigeria.^15^ The AR were also lower than the AR values reported for *fufu* made with oven‐dried fermented cassava flour (17–32%) in Nigeria.^14^ The lower carotenoid retention in *fufu* could be due to the exposure of carotenoids to sunlight during drying, as observed by Chávez *et al*.,^6^ who reported higher carotenoid TR (71.9%) when drying cassava with an oven compared with sun‐drying (37.9%). Fermentation and pressing was the step during preparation of *fufu* that produced the highest pVACs loss (56.2%), followed by 40.1% loss during sun‐drying of fermented *microcossettes*. The cooking step caused only 1.7% of pVACs loss (Fig. 1).

In DRC and the Republic of Congo, fermented cassava to prepare *fufu* is generally dried as large chips (*cossettes*), which are large pieces of roots. To minimize product losses due to molding during drying, the use of smaller chips (*microcossettes*) to give fast drying is widely promoted. Given the low pVACs retention observed in this study using *microcossettes*, it will be important to study the rate of carotenoid loss when sun‐drying large pieces of cassava and determine if higher carotenoid retention could be obtained compared with the currently suggested method using *microcossettes*.

### Carotenoid retention in cooked *chikwangue*

Large variability in pVACs TR between genotypes was observed when producing *chikwangues*. The TR values of carotenoids in cooked *chikwangue* prepared with freshly fermented cassava were 4.0–18.1%. Genotype Vuvu had the highest TR, but its AR was not significantly different to the other genotypes. The pVACs in cooked *chikwangue* was 0.52–1.75 µg g^−1^ FW and 1.39–6.73 µg g^−1^ DW (Table 2). Significant carotenoids loss occurred in each processing step for *chikwangue*. On average, the greatest loss of pVACs was during the production of the fine paste (75.7%), whereas during cooking cassava paste into *chikwangue* the loss was 15.4% (Fig. 1).

Higher pVACs loss was found during production of *chikwangue* paste than with *fufu* paste, except for the genotype Vuvu. This difference could be attributed to the higher dry matter loss when producing paste for *chikwangue* (70.5%) compared with *fufu* (36.7%). The higher dry matter loss in *chikwangue* paste was due to the additional sieving step in which 16.8–40.6% of dry matter was removed as fine residue. The pVACs in the residue removed during sieving represented 3.8–21.6% of the initial pVACs and significantly increased the pVACs loss in *chikwangue* paste (*p* < 0.05). The amount of residue removed was positively correlated to the total amount of pVACs retained in the residue (*r* ^2^ = 0.95), but the proportion of pVACs loss was lower than the proportion of solids loss.

In general, pVACs TR in *chikwangue* was lower than boiled cassava but higher than *fufu* (Fig. 1). Despite the low pVACs TR of *chikwangue* (8.9%), its AR was high (28.1%) compared with *fufu* (3.2%). Cooked *chikwangue* could deliver a higher amount of pVACs than cooked *fufu* when considering daily consumption of final cooked product only, without accounting for dry matter losses during processing. Considering the significant carotenoid losses during sieving, biofortified cassava varieties that produce lower residue during *chikwangue* preparation should be developed.

### Relationship of carotenoids by HPLC and spectrophotometry

**Figure 2:**
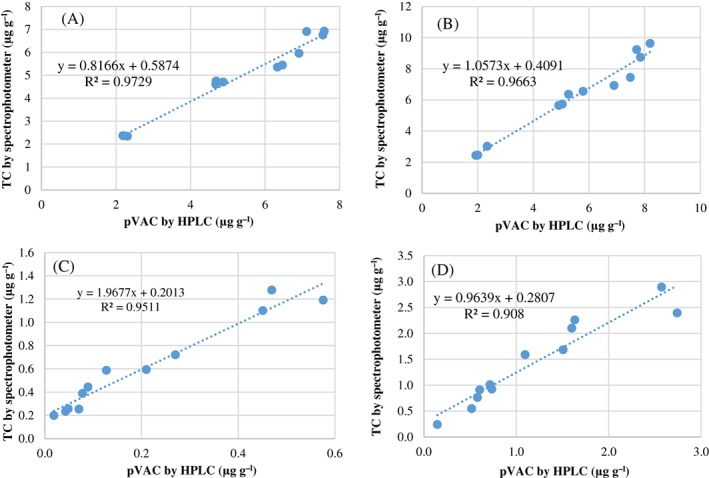
Correlation between carotenoids determined by HPLC and spectrophotometry for (A) fresh peeled roots, (B) boiled roots, (C) cooked *fufu*, and (D) cooked *chikwangue* prepared with cassava roots.

In fresh roots, TCs values measured by spectrophotometry and pVACs values by HPLC had a high correlation (*r* ^2^ = 0.97). The correlation was also high for boiled roots (*r* ^2^ = 0.97), cooked *fufu* (*r* ^2^ = 0.95), and cooked *chikwangue* (*r* ^2^ = 0.91) (Fig. 2). The high correlation for raw cassava roots is in concordance with Sánchez *et al*.,^23^ who suggested that spectrophotometry is a reliable alternative to measure TCs for fast genotyping cassava in breeding programs, instead of HPLC. Given that no major changes in proportions of 13‐*cis‐β*C and 9‐*cis‐β*C were found between raw and cooked cassava, the high correlation found for raw cassava was also expected for cooked products. The use of spectrophotometry to estimate pVACs content in processed products should be considered as a tool to select material during the breeding process, not only to analyze raw and boiled cassava, but also for processed products such as *fufu* and *chikwangue*. The significant genotypic effect on carotenoid retention during processing and limited availability of equipment (such as for HPLC) in countries where biofortified breeding programs exist are reasons to consider the use of spectrophotometry to estimate pVACs content in raw and processed roots in breeding programs, where large numbers of genotypes are evaluated.

### Estimation of degradation kinetics of carotenoids in cassava paste and *microcossettes*

Considering that fermented cassava *microcossettes* and paste are commonly used for cooking up to 1 month after processing, an estimation of the TC degradation kinetics during common storage for such products was determined based on measurements of retention values at different timepoints. TC retention in *microcossettes* was 62 ± 17% after 20 days of storage and 65 ± 10% for paste stored for 30 days. A first‐order degradation was observed for TC in both products for biofortified Kindisa variety processed in seven locations in Kongo Central province. The degradation rate constant *k* (day^−1^) was higher in fermented cassava *microcossettes* than in cassava paste, with averages of 0.0282 (*r* ^2^ = 0.73) and 0.0144 (*r* ^2^ = 0.89) respectively (*p* < 0.01). The *k* values for TC in cassava *microcossettes* were similar to values reported for *β*C in dry cassava granules stored at 26–33 °C (*k* = 0.0144–0.0271) by Bechoff *et al*.^24^ Retention values after 30 days at such degradation rates represented 65.0% for fermented *chikwangue* paste and 43.0% for fermented cassava *microcossettes*, at 24–27 °C. It is known that the rate of degradation of pVACs is relatively high in dry products, especially when the particle size of the product is small, due to its high surface area exposing the carotenoids to oxygen.^25^, ^26^, ^27^ Lower *k* values for fermented cassava paste suggest that carotenoids in an aqueous matrix are more stable than carotenoids exposed to oxygen in a dry matrix such as cassava *microcossettes*. These results are in concordance with previous studies showing that the main factor influencing degradation of carotenoids during storage of biofortified foods is oxygen availability.^28^, ^29^ Assuming that products are consumed in similar proportion during a 30 day storage period, the resulting average TCs retention for *fufu* and *chikwangue* could be 68% and 82% respectively.

### Contribution of biofortified cassava to improve vitamin A intake

The contribution of biofortified cassava to vitamin A intake in populations with high cassava consumption as *fufu* and *chikwangue* was estimated for all genotypes. Cassava processed as *fufu* had significantly higher pVACs loss than boiled cassava for each genotype, whereas *chikwangue* had less pVACs loss than *fufu* but more than boiled cassava. These differences in TR have an impact on the vitamin A EAR contribution from cassava based on type of product eaten. Contribution to the EAR was calculated considering an EAR of 275 µg for children aged 4–6 years and 500 µg for women of child‐bearing age, as suggested by the Institute of Medicine.^30^ Average TC storage retention, 68% for *fufu* and 82% for *chikwangue*, was used based on the TC storage retention obtained for Kindisa variety. Intake of 300 g of peeled cassava for children and 500 g for women was used based on cassava intake from Nigeria and production in DRC.^1^, ^31^ For children aged 4–6 years, biofortified cassava would contribute 117%, 8%, or 2% of the vitamin A requirement when respectively consuming boiled cassava, *chikwangue*, or fermented *fufu* prepared with 300 g of peeled cassava roots (Table 3). For women of child‐bearing age, biofortified cassava would contribute 107%, 8%, or 2% of the EAR when 500 g of peeled cassava is eaten as boiled, *chikwangue*, or *fufu* respectively (Table 3). The potential contribution to the EAR (280–305%) by consuming only boiled cassava prepared with a variety containing the pVACs target level (15 µg g^−1^) may not be reached in countries where *fufu* and *chikwangue* are the main cassava‐based products. However, with a partial substitution of 10%, the contribution of biofortified boiled cassava to the EAR could be up to 31% for children 4–6 years old and 28% for women, higher than when *chikwangue* and *fufu* are consumed as the only source of cassava product (Table 3). Using 17% and 18% of biofortified cassava with the full pVACs target content to prepare boiled cassava could provide enough pVACs to reach >50% of the vitamin A EAR for children 4–6 years old and women respectively (Table 3). Cyanide values reported for biofortified cassava are not always lower than recommended for boiled cassava.^23^ To promote consumption of biofortified cassava as boiled, breeders need to develop biofortified cassava varieties with low levels of cyanide to reduce the risk of toxicity, and farmers should be trained on proper boiling methods to reduce cyanide content.^22^

**Table 3:** Estimation of the contribution of four cassava genotypes and target variety (15 µg g^−1^ pVACs) to vitamin A estimated average requirement (EAR) for children aged 4–6 years and women when cassava prepared as boiled, *chikwangue*, or *fufu* at different usage levels

Product	Usage (%)	Population	EAR (%)(a ^,^ b)				
			Vuvu	MVZ2012/044	Kindisa	MVZ2011B/360	Target
Boiled	100	Children 4–6	37	87	117	140	305
	100	Women	34	80	107	129	280
	10	Children 4–6	4	9	12	14	31
	10	Women	3	8	11	13	28
	17	Children 4–6	6	15	20	24	52
	18	Women	6	14	19	23	50
*Chikwangue*	100	Children 4–6	6	3	8	7	11
	100	Women	5	3	8	6	10
	20	Children 4–6	1	1	2	1	2
	20	Women	1	1	2	1	2
*Fufu*	100	Children 4–6	1	2	1	2	2
	100	Women	1	2	1	2	2
	70	Children 4–6	0	1	1	1	1
	70	Women	0	1	0	1	1

a EAR, estimated average requirement. EAR for children 4–6 = 275 µg retinol equivalents (RE) and for women = 500 µg RE.

b Calculated based on the lowest retention for each type of product within the 4 genotypes tested.

In general, the TR of carotenoids was high in boiled cassava, low in *chikwangue*, and very low in *fufu* using current processing methods in DRC and the Republic of Congo. Promoting the consumption of *chikwangue* and boiled cassava instead of *fufu* could be a good strategy, especially if considering only the concentration of pVACs in the final product (apparent retention) without accounting for the losses of dry matter during processing. Boiled cassava has the potential to achieve the 50% EAR of vitamin A target level for biofortified crops; however, consuming biofortified cassava only as *fufu* and *chikwangue* may not provide the vitamin A intake target level. Since consumers in rural areas of DRC and the Republic of Congo eat less *chikwangue* than *fufu*, farmers could produce *chikwangue* with biofortified yellow cassava and *fufu* with white cassava. The consumption of boiled products in areas where high deficiency of vitamin A exists should be promoted only if the cassava roots used have low levels of cyanide. Meanwhile, *fufu* and *chikwangue* processing should be improved to minimize carotenoid loss, not only in African countries, but in any country where the processing includes extensive loss of dry matter and sun‐drying. Considering the genotypic variation for pVACs retention during processing, it is recommended to evaluate the carotenoid retention for *chikwangue* and *fufu* made with advanced biofortified breeding material before they are released as new varieties. Spectrophotometry or HPLC methods could be used in such breeding programs to test raw cassava and *fufu* and *chikwangue*.
